# High expression of Talin-1 is associated with tumor progression and recurrence in melanoma skin cancer patients

**DOI:** 10.1186/s12885-023-10771-z

**Published:** 2023-04-03

**Authors:** Yasaman Rezaie, Fahimeh Fattahi, Baharnaz Mashinchi, Kambiz Kamyab Hesari, Sahar Montazeri, Elham Kalantari, Zahra Madjd, Leili Saeednejad Zanjani

**Affiliations:** 1 Oncopathology Research Center, Iran University of Medical Sciences (IUMS), Hemmat Street (Highway), Next to Milad Tower, Tehran, 14496-14535 Iran; 2 School of Medicine, Tehran University of Medical Sciences, Tehran, Iran; 3 Department of Dermatopathology, Razi Hospital, Tehran University of Medical Sciences (TUMS), Tehran, Iran; 4 Department of Pathology and Genomic Medicine, Sidney Kimmel Cancer Center, Thomas Jefferson University, Philadelphia, PA USA

**Keywords:** Talin-1, Skin cancer, Melanoma, Non-melanoma skin cancer, Bioinformatics, IHC

## Abstract

**Background:**

Talin-1 as a component of multi-protein adhesion complexes plays a role in tumor formation and migration in various malignancies. This study investigated Talin-1 in protein levels as a potential prognosis biomarker in skin tumors.

**Methods:**

Talin-1 was evaluated in 106 skin cancer (33 melanomas and 73 non-melanomas skin cancer (NMSC)) and 11 normal skin formalin-fixed paraffin-embedded (FFPE) tissue samples using immunohistochemical technique on tissue microarrays (TMAs). The association between the expression of Talin-1 and clinicopathological parameters, as well as survival outcomes, were assessed.

**Results:**

Our findings from data minings through bioinformatics tools indicated dysregulation of Talin-1 in mRNA levels for skin cancer samples. In addition, there was a statistically significant difference in Talin-1 expression in terms of intensity of staining, percentage of positive tumor cells, and H-score in melanoma tissues compared to NMSC (P = 0.001, P < 0.001, and P < 0.001, respectively). Moreover, high cytoplasmic expression of Talin-1 was found to be associated with significantly advanced stages (P = 0.024), lymphovascular invasion (P = 0.023), and recurrence (P = 0.006) in melanoma cancer tissues. Our results on NMSC showed a statistically significant association between high intensity of staining and the poor differentiation (P = 0.044). No significant associations were observed between Talin-1 expression levels and survival outcomes of melanoma and NMSC patients.

**Conclusion:**

Our observations showed that higher expression of Talin1 in protein level may be significantly associated with more aggressive tumor behavior and advanced disease in patients with skin cancer. However, further studies are required to find the mechanism of action of Talin-1 in skin cancers.

**Supplementary Information:**

The online version contains supplementary material available at 10.1186/s12885-023-10771-z.

## Background

Skin cancers are the most common neoplasms in humans, imposing high rates of disease burden globally [1, 2]. They are generally classified as melanoma and non-melanoma skin cancers (NMSC), with squamous cell carcinoma (SCC) and basal cell carcinoma (BCC) being the most prevalent non-melanoma subtypes [3]. NMSCs are responsible for most skin cancer cases; however, melanoma is the leading cause of death due to skin cancer and one of the most aggressive cancers [4–6], with an estimation of 99,780 new cases and 7650 deaths in 2022 in the United States alone [7]. NMSCs have a relatively low mortality risk, however, due to their high prevalence, they impose a high burden on public health [3, 6, 8, 9]. Although melanoma comprises only 1% of skin cancers, it has a reputation for rapid metastasis and resistance to therapy, and the mechanisms of cellular invasion remain mostly unclear [10, 11]. Early-stage melanoma is easily curable with excision, however, with metastasis, the survival rates decrease drastically [12]. Despite many new treatment options for advanced melanoma today, the response to treatment differs in patients, and many patients may develop resistance to therapy or adverse effects [13–15]. Diagnostic and prognostic biomarkers are increasingly important in melanoma treatment so that they may have improved survival and treatment [16–18]. In clinical settings, proper prognostication of patients is necessary to select the suitable treatment plan for each patient to maximize efficacy and decrease the expenses and adverse effects of the treatment. Although many new genomic and protein markers including the S-100b and Lactate dehydrogenase (LDH) proteins have been suggested for advanced melanoma prognostication, no feasible lab tests are available today to stratify high-risk patients with high accuracy in clinical settings [19, 20]. New prognostic markers and therapeutic targets for both melanoma and NMSCs are urgently needed to optimize patient management and decrease the morbidity and mortality of the disease [18].

Talin-1 is a large protein interacting with the cytoplasmic domain of the integrin β subunit, connecting it to focal adhesion molecules such as focal adhesion kinase (FAK) and vinculin and regulatory molecules, including deleted in liver cancer-1 (DLC-1), RIAM and KANK [21–25]. As the main component of focal adhesions (FAs), Talin-1 links the extracellular matrix (ECM) and actin cytoskeleton, acting as a mechanosensitive signaling hub regulating the cell behavior such as proliferation, migration, and cell shape according to changes in the ECM [26–31]. In healthy skin tissue, Talin-1 is present at the epidermal-dermal interface and in cell-cell junctions of the melanocytes, emphasizing its crucial function in cell-cell and cell-ECM adhesion and signaling [32, 33].

The role of Talin-1 dysregulation in cancer has been studied widely; however, a substantial controversy in the literature exists today regarding the regulation of Talin-1 in different cancers. Upregulation of Talin-1 expression is documented in gastric cancer, mucosal SCC, and prostate cancers; conversely, its downregulation is shown in colorectal cancer and hepatocellular carcinoma [34–39]. High expression levels of Talin-1 correlated with invasion and lower survival rates in prostate cancer, colon cancer, nasopharyngeal carcinoma, and oral SCC [35, 40–42]. Furthermore, Talin-1 knockdown in prostate cancer and colorectal cancer cell lines has been shown to reduce their migration and proliferation [43, 44].

The role and clinical significance of Talin-1 protein in melanoma and NMSCs remain unexplored. In the present study, at the primary search stage, comprehensive alterations in mRNA levels of Talin-1 in patients with skin cancer were analyzed using Gene Expression Profiling Interactive Analysis (GEPIA2) and Gene Expression database of Normal and Tumor tissues 2 (GENT2) databases. Then, Talin-1 protein expression levels were evaluated by formalin-fixed paraffin-embedded (FFPE) tissue samples which were assembled on tissue microarray (TMA) slides using Immunohistochemical (IHC) technique. The IHC assay is a standard procedure applied to assess novel molecular biomarkers, and TMA technology enables simultaneous staining of hundreds of tissue samples [45]. Then, we sought to determine the association of Talin-1 expression with clinicopathological characteristics and survival information of skin cancer patients.

## Methods

### **Investigations of Talin-1 based on data mining**

To investigate alteration in the expression of Talin-1 in mRNA levels for patients with skin cancer, GEPIA2 and GENT2 databases were applied. GEPIA2 (http://gepia2.cancer-pku.cn/) is an online database using RNA expression data of tumor and normal samples obtained from The Cancer Genome Atlas (TCGA) and the Genotype-Tissue Expression (GTEx) [46]. While GENT2 (http://gent2.appex.kr) is included gene expression data of cancer and normal tissues from the NCBI-GEO database with two microarray platforms (Affymetrix U133A or U133Plus2) [47]. Therefore, boxplot mRNA expression analysis for skin cancer and normal tissues was constructed through these databases. Moreover, the UCSC Xena Browser database (https://xenabrowser.net/) was used to evaluate expression data with metastasis feature in RNA-sequencing data for Talin-1 expression from skin cancer patients. UCSC Xena Browser is an online visual exploration tool for public and private, multi-omic, and clinical/phenotype data including TCGA and Genomic Data Commons (GDC) [48]. Finally, a bioinformatics analysis of the survival data as it relates to Talin-1 in mRNA levels for skin cancer patients was performed using these mentioned online databases.

### Study population

A total of 106 FFPE archival specimens of skin cancer including melanoma skin cancer (N = 33), NMSC consisting of SCC and BCC (N = 73), and normal skin tissues (N = 11) were collected from Razi referral skin hospital and Imam Khomeini tertiary complex in Tehran, Iran. Samples were obtained from patients between the years 2013–2020, and it was confirmed that none of the patients were subject to chemotherapy or radiotherapy prior to tissue sampling. Hematoxylin and eosin (H&E) stained slides associated with each FFPE sample were collected for TMA construction. We reviewed patients’ medical records for clinicopathological characteristics including gender, age, TNM stage, histologic grade, Breslow thickness, ulceration, lymphovascular invasion (LVI), perineural invasion (PNI), lymphocyte infiltration, distant metastasis, and tumor recurrence. Next, patients’ survival information was gathered by telephone follow-ups. The time between the initial treatment and death due to skin cancer was defined as disease-specific survival (DSS), and progression-free survival (PFS) was defined as the interval between the initial treatment and the last follow-up without evidence of disease progression and metastasis. The TNM stage was determined according to the American Joint Committee on Cancer (AJCC) [49]. All patients’ information was handled with confidentiality, and the research process was approved by the medical ethics committee of Tehran University of medical sciences under the code IR.TUMS.REC.1401.034.

### TMA construction

Skin cancer TMAs were constructed as described in the literature [50, 51]. Briefly, our pathologists (SM and KK) evaluated the H&E stained slides and FFPE blocks and marked the most representative area of the tumor. The selected spots were punched by a precision arraying instrument (Tissue Arrayer Minicore; ALPHELYS, Plaisir, France), and tissue cylinders were separated and transferred to the recipient block. For more accurate results, TMA blocks were triplicated, and from each block, separate slides were constructed and stained. The final staining score of each sample was determined by the mean staining score of the cores.

### Immunohistochemical (IHC) staining

First, all TMA slides were deparaffinized for 20 min and were transferred in xylene; then, the rehydration process was carried out via serial-graded alcohols. To block the endogenous peroxidase activity and prevent non-reactive staining, we used a 3% hydrogen peroxide solution for 20 min at room temperature. After washing the slides, we autoclaved the samples in citrate buffer (ph = 6.0) for 10 min to retrieve sample antigens. Next, we blocked sections (blocker protein, Dako, Denmark) for 20 min and incubated them with a primary rabbit polyclonal antibody to Talin-1 (1:500, ab71333, Abcam Inc., Cambridge, MA, UK) at 4 °C overnight. The next day, slides were washed three times with Tris-buffered saline (TBS) and incubated with anti-rabbit/anti-mouse envision IgG–HRPO (EnVision, Dako, Denmark) as the secondary antibody for 1 h. Afterward, TMA slides were treated for 3 min at room temperature with 3,3′-diaminobenzidine (DAB) (Dako, Denmark) substrate as a chromogen. Finally, slides were counterstained with hematoxylin (Dako, Denmark), dehydrated with serial-graded alcohol, cleared with xylene, and mounted. Human normal kidney tissue was used as a positive control, and for negative control samples, the primary antibody was replaced with TBS to ensure no nonspecific bindings happened. The optimal dilution of the Talin-1 antibody was examined by applying serial-prepared dilutions of the antibody to the tissue.

### Immunostaining assessment

Two experienced dermatopathologists (SM and KK) independently evaluated immunostained sections blinded to patients’ information. Any inconsistency in results was resolved by reaching a consensus. The intensity and area of staining were the primary reported outcomes. The intensity of staining was reported in a semi-quantitative fashion, represented by 0 (no staining), 1 (weak), 2 (moderate), and 3 (strong). The staining area was reported as the percentage of positive tumor cells in the sample and classified into four groups: <25%, 25–50%, 51–75%, and > 75%. We reported the final Talin-1 expression results as histochemical score (H-score), calculated by multiplying the staining intensity by the staining area, ranging from 0 to 300. In this study, the median H-score was used to categorize the samples as expressing high or low levels of talin-1.

### Statistical analysis

All patients’ data and scoring results were documented and analyzed using “statistical software SPSS, version 22.0. Armonk, NY: IBM Corp”. We reported the categorical data by *N* (%), valid percent, and quantitative data as follows: mean (SD) and median (Q1, Q3). First, the Mann-Whitney *U* test was applied to compare the significance of staining differences in melanoma and NMSC tissues. Afterward, Pearson’s chi-square test and Spearman’s correlation tests were used to analyze the significance of association and correlation between Talin-1 expression and clinicopathological characteristics of patients. Ultimately, we analyzed survival data regarding Talin-1 expression by the Kaplan-Meier method with a 95% confidence interval and compared the results with the log-rank test. The Cox proportional hazards regression model was adopted to perform univariate and multivariate analyses. Any differences with a p-value < 0.05 were considered significant.

## Results

### Data mining

**Fig. 1:**
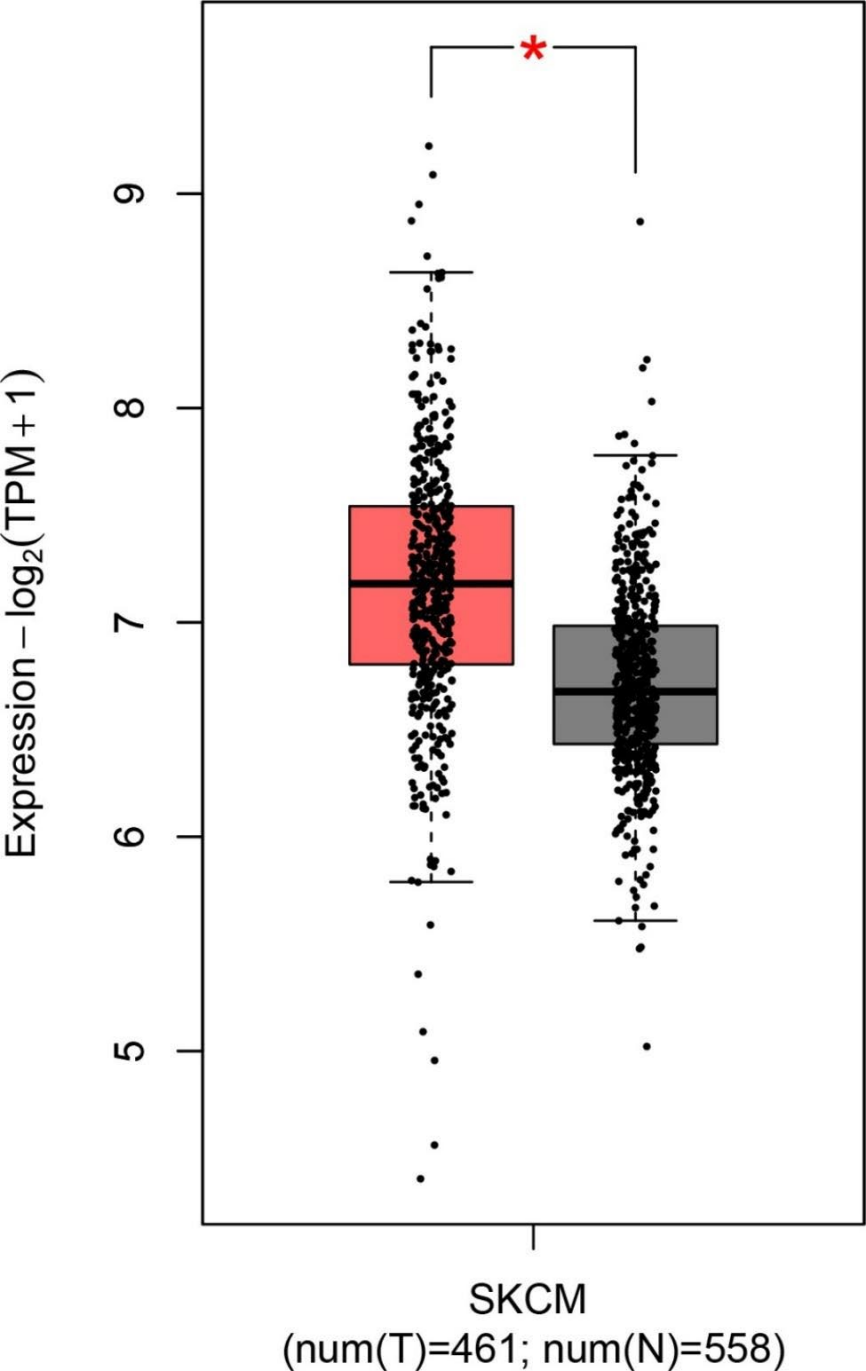
**The mRNA levels of Talin-1 (TLN1) in Skin Cutaneous Melanoma using Gene** **Expression Profiling Interactive Analysis 2 (GEPIA2). **High expression of Talin-1 was found in mRNA levels in tumors compared with normal tissues (|Log2FC| Cut-off ≥ 0.5 and P < 0.01) by Gene Expression Profiling Interactive Analysis (GEPIA2).

**Fig. 2:**
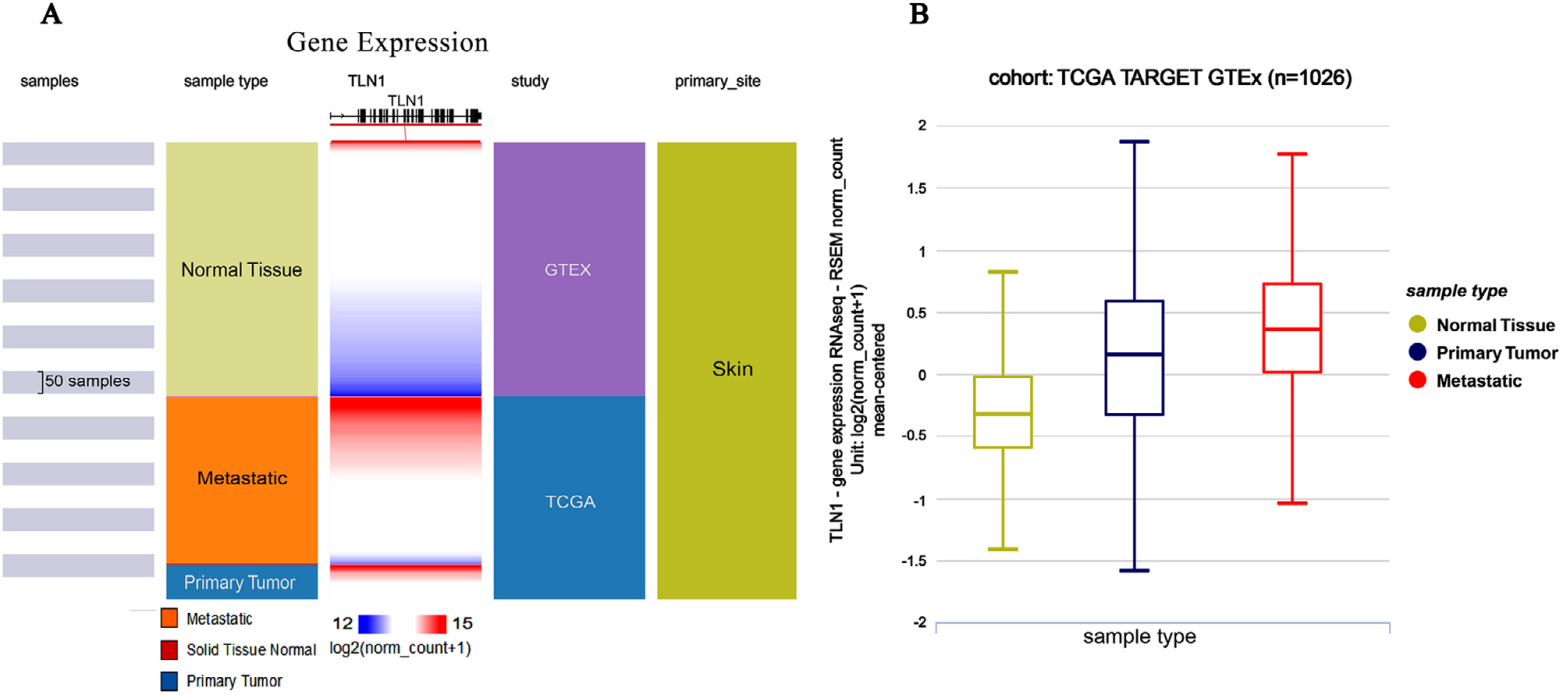
**Comparison between Talin-1 (TLN1) expression from normal tissues, primary tumors, and metastatic tissues for skin data on UCSC Xena web-based tool. (A)** heat map and **(B)** boxplot showed a high expression levels of Talin-1 in melanoma skin cancer patients with metastases rather than primary tumor tissues (Welch’s t-test, P = 0.008 (t = -2.679))

The results of the TCGA database via GEPIA2 revealed that mRNA expression of the Talin-1 gene was higher (|Log2FC| Cut-off ≥ 0.5) in 461 skin cutaneous melanoma tissues compared to the 558 normal skin tissues (P < 0.01, Fig. 1). Furthermore, obtained data based on GENT2 from the displayed GEO mRNA expression level of Talin-1 was significantly higher in skin cancer tissues compared to the normal skin tissues (GPL570 platform) (Supplementary Tables 1 and Supplementary Fig. 1). After analyzing of the skin cancer patient data from the TCGA database using the UCSC Xena web-based tool, a higher expression of Talin-1 was observed in skin cancer patients with metastases (n = 366) compared to primary tumor tissues (n = 102) (Welch’s t-test, p = 0.008 (t = -2.679)). The comparison between gene expression of data from normal tissues, primary tumors, and metastatic tissues was shown in the heat map and boxplot in Fig. 2. Although no significant data was found in the evaluation of prognosis biomarkers from Talin-1 at RNA level through survival analysis.

### Demographics of skin cancer patients

Following the IHC staining of tissue samples, a total of 106 archival FFPE samples as TMA slides were included in the study, of which 33 (31.1%) were melanoma skin cancer and 73 (68.7%) were NMSC tissues (34 BCC and 39 SCC samples). Eleven samples of normal skin tissues were used as controls. Melanoma samples belonged to 18 (54.5%) male and 15 (45.5%) female patients with a mean age of 65 ± 13.9 (range: 35–91). NMSC samples were obtained from 57 (78.0%) male and 16 (21.9%) female patients with a mean age of 68.4 ± 1.34 (range:38–94). Clinicopathological characteristics of melanoma and NMSC patients are demonstrated in Supplementary Tables 2 and 3, respectively.

### Expression of Talin-1

#### Comparison of Talin-1 expression in melanoma and non-melanoma skin cancers (NMSC) tissue samples

**Fig. 3:**
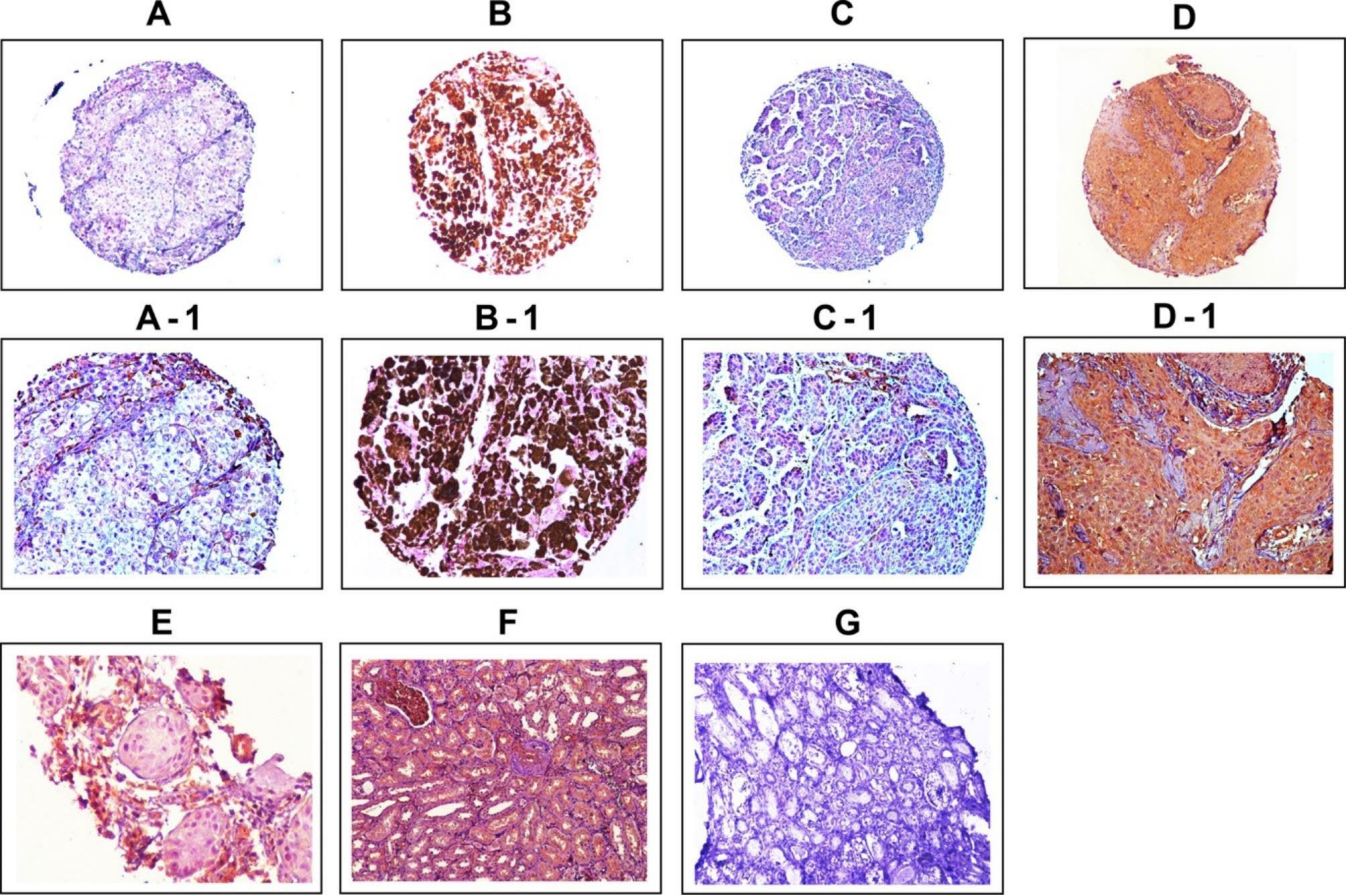
**Immunohistochemical staining of Talin-1 protein expression in skin cancer and normal skin tissues.** Talin-1 protein expression in melanoma skin cancer tissues: low expression **(A, A-1)** and high expression **(B, B-1)**. Talin-1 protein expression in non melanoma skin cancer (NMSCs) tissues low expression **(C, C-1)**, and high expression **(D, D-1)**. IHC staining of skin normal tissue **(E)**, and human normal kidney as **(F)** positive and **(G)** negative controls. Figures are shown with a magnification of 100 × and 200×

The expression level of Talin-1 in skin cancer tissues was evaluated through the IHC method on TMA sections by measuring the intensity of staining, area of staining, and H-score. All cores showed different levels of staining in the cytoplasm (Fig. 3). We divided samples into low expression and high expression groups according to the median cytoplasmic expression of H-scores (cutoff = 200 and 90 in melanoma and NMSC tissues, respectively). The results showed that there is a statistically significant difference in Talin-1 expression in terms of intensity of staining, percentage of positive tumor cells, and H-score in melanoma tissues compared to NMSC samples (P = 0.001, P < 0.001, and P < 0.001, respectively) (Table 1). Higher expression levels of Talin-1 were observed in skin tumor tissues compared to normal tissue samples (melanoma and NMSC tissues). Moreover, normal human kidney tissue used as a positive control showed strong staining in renal epithelial cells (Fig. 3).

**Table 1:** Expression of Talin-1 (Intensity of staining, percentage of positive tumor cells, and H-score) in melanoma skin cancer tissues, non-melanoma skin cancer, and normal skin tissues

Scoring system	Melanoma skin cancer tissues N (%)	Non-melanoma skin cancer tissues N (%)	*P-value*	Normal skin tissues N (%)
Intensity of staining Negative (0) Weak (+ 1) Moderate (+ 2) Strong (+ 3)	0 (0.0) 10 (30.3) 18 (54.5) 5 (15.2)	0 (0.0) 49 (67.1) 19 (26.0) 5 (6.8)	***0.001***	0 (0.0) 7 (63.6) 4 (36.4) 0 (0.0)
Percentage of positive tumor cells < 25% 25–50% 51–75% > 75%	0 (0.0) 1 (3.0) 2 (6.1) 30 (90.9)	12 (16.4) 8 (11.0) 13 (17.8) 40 (54.8)	***< 0.001***	2 (18.2) 1 (9.1) 2 (18.2) 6 (54.5)
H-score cut off Low High	200 29 (87.9) 4 (12.1)	90 42 (57.5) 31 (42.5)	***< 0.001***	4 (36.4) 7 (63.6)
Total	33	73		11

*H-score* histological score

*P value* is based on Mann-Whitney *U* test

#### Associations of Talin-1 expression and clinicopathological characteristics in melanoma tissues

We collected patients’ data, including age, gender, TNM stage, Breslow thickness, ulceration, LVI, PNI, lymphocytic infiltration, distant metastasis, and tumor recurrence. Pearson’s χ2 test was utilized to find the association of Talin-1 expression with clinicopathological features. Our findings indicated that there is a statistically significant association between high expression levels of Talin-1 and advanced TNM stage (Hscore P = 0.024), LVI (intensity of staining P = 0.024; H-score P = 0.023) as well as tumor recurrence (Hscore P = 0.006). No significant associations were detected between Talin-1 expression and other clinicopathological parameters (Table 2). Moreover, Bivariate analysis showed a statistically significant positive correlation between increased Talin-1 expression and increase in TNM stage (Spearman’s rho, P = 0.025), and between high Talin-1 expression and LVI (P = 0.023) as well as tumor recurrence (P = 0.005).

**Table 2:** The association between expression of Talin-1 and clinicopathological parameters of melanoma skin cancer tissues (Intensity of staining and H-score) (P value; Pearson’s χ2 test)

Tumor characteristics	Total samples N (%)	Intensity of staining N (%)				*P-value*	H-score (cut off = 200) N (%)		*P-value*
		0 (Negative)	1+ (Weak)	2+ (Moderate)	3+ (Strong)		Low (≤ 200)	High (> 200)	
Mean age, years (Range) ≤ Median age > Median age	45 (16–74) 18 (39.1) 28 (60.9)	0 (0.0) 0 (0.0)	7 (21.2) 3 (9.1)	8 (24.2) 10 (30.3)	3 (9.1) 2 (6.1)	0.413	15 (51.7) 14 (48.3)	3 (75.0) 1 (25.0)	0.381
Gender Male Female	18 (54.5) 15 (45.5)	0 (0.0) 0 (0.0)	5 (15.2) 5 (15.2)	10 (30.3) 8 (24.2)	3 (9.1) 2 (6.1)	0.927	16 (48.5) 13 (39.4)	2 (6.1) 2 (6.1)	0.846
TNM stage I II III IV	3 (9.1) 3 (9.1) 2 (6.1) 25 (75.8)	0 (0.0) 0 (0.0) 0 (0.0) 0 (0.0)	0 (0.0) 1 (3.0) 1 (3.0) 8 (24.2)	1 (3.0) 2 (6.1) 0 (0.0) 15 (45.5)	2 (6.1) 0 (0.0) 1 (3.0) 2 (6.1)	0.91	1 (3.0) 3 (9.1) 2 (6.1) 23 (69.7)	2 (6.1) 0 (0.0) 0 (0.0) 2 (6.1)	***0.024***
Breslow thickness (Range) < 1 1–4 4 >	3 (21.4) 5 (35.7) 6 (42.9)	0 (0.0) 0 (0.0) 0 (0.0)	0 (0.0) 1 (7.1) 1 (7.1)	1 (7.1) 3 (21.4) 4 (28.6)	2 (14.3) 1 (7.1) 1 (7.1)	0.572	1 (7.1) 5 (35.7) 5 (35.7)	2 (14.3) 0 (0.0) 1 (7.1)	0.078
Ulceration Yes No	10 (38.5) 16 (61.5)	0 (0.0) 0 (0.0)	1 (3.8) 6 (23.1)	7 (26.9) 8 (30.8)	2 (7.7) 2 (7.7)	0.304	8 (30.8) 15 (57.7)	2 (7.7) 1 (3.8)	0.286
Lymphovascular invasion (LVI) Yes No	10 (58.8) 7 (41.1)	0 (0.0) 0 (0.0)	3 (17.6) 1 (5.9)	7 (41.2) 2 (11.8)	0 (0.0) 4 (23.5)	***0.024***	10 (58.8) 4 (23.5)	0 (0.0) 3 (17.6)	***0.023***
Perineural invasion (PNI) Yes No	4 (16.6) 20 (83.3)	0 (0.0) 0 (0.0)	1 (4.2) 6 (25.0)	2 (8.3) 11 (45.8)	1 (4.2) 3 (12.5)	0.885	3 (12.5) 18 (75.0)	1 (4.2) 2 (8.3)	0.408
Lymphocyte infiltration Yes No	12 (75.0) 4 (25.0)	0 (0.0) 0 (0.0)	2 (12.5) 2 (12.5)	7 (43.8) 2 (12.5)	3 (18.8) 0 (0.0)	0.306	10 (62.5) 4 (25.0)	2 (12.5) 0 (0.0)	0.383
Distant metastasis Yes No	25 (75.8) 8 (24.2)	0 (0.0) 0 (0.0)	8 (32.0) 2 (6.1)	15 (45.5) 3 (9.1)	2 (6.1) 3 (9.1)	0.126	23 (69.7) 6 (18.2)	2 (6.1) 2 (6.1)	0.200
Tumor recurrence Yes No	24 (85.7) 4 (14.2)	0 (0.0) 0 (0.0)	8 (28.6) 0 (0.0)	14 (50.0) 2 (7.1)	2 (7.1) 2 (7.1)	0.063	23 (82.1) 2 (7.1)	1 (3.6) 2 (7.1)	***0.006***

* H-score* histological score

Values in bold are statistically significant

#### Associations of Talin-1 expression and clinicopathological characteristics in non-melanoma skin cancers (NMSC) tissues

The results of Pearson’s χ2 test exhibited no significant associations between Talin- 1 expression and clinicopathological parameters, including age, gender, TNM stage, histologic grade, ulceration, lymphocyte infiltration, distant metastasis, and recurrence in NMSC tissues, except that high expression of Talin-1 in terms of intensity of staining was associated with an increase in histologic grade in NMSC tissues (P = 0.044) (Table 3).

**Table 3:** The association between expression of Talin-1 and clinicopathological parameters of non-melanoma skin cancer (NMSC) tissues (Intensity of staining and H-score) (P-value; Pearson’s χ2 test)

Tumor characteristics	Total samples N (%)	Intensity of staining N (%)				*P-value*	H-score (cut off = 90) N (%)		*P-value*
		0 (Negative)	1+ (Weak)	2+ (Moderate)	3+ (Strong)		Low (≤ 90)	High (> 90)	
Mean age, years (Range) ≤ Median age > Median age	45 (16–74) 38 (52.1) 35 (47.9)	0 (0.0) 0 (0.0)	26 (35.6) 23 (31.5)	9 (12.3) 10 (13.7)	3 (4.1) 2 (2.7)	0.855	22 (30.1) 20 (27.4)	16 (21.9) 15 (20.5)	0.948
Gender Male Female	57 (78.1) 16 (21.9)	0 (0.0) 0 (0.0)	36 (49.3) 13 (17.8)	17 (23.3) 2 (2.7)	4 (5.5) 1 (1.4)	0.357	33 (45.2) 9 (12.3)	24 (32.9) 7 (9.6)	0.906
TNM stage* I II III IV	17 (43.6) 15 (38.5) 2 (5.1) 5 (12.8)	0 (0.0) 0 (0.0) 0 (0.0) 0 (0.0)	10 (25.6) 8 (20.5) 1 (2.6) 3 (7.7)	7 (17.9) 6 (15.4) 1 (2.6) 1 (2.6)	0 (0.0) 1 (2.6) 0 (0.0) 1 (2.6)	0.701	10 (25.6) 8 (20.5) 1 (2.6) 4 (10.3)	7 (17.9) 7 (17.9) 1 (2.6) 1 (2.6)	0.758
Histological grade* Well Moderate Poor	21 (53.8) 13 (33.3) 5 (12.8)	0 (0.0) 0 (0.0) 0 (0.0)	16 (41.0) 4 (10.3) 2 (5.1)	5 (12.8) 7 (17.9) 3 (7.7)	0 (0.0) 2 (5.1) 0 (0.0)	***0.044***	15 (38.5) 6 (15.4) 2 (5.1)	6 (15.4) 7 (17.9) 3 (7.7)	0.226
Ulceration Yes no	15 (34.1) 29 (65.9)	0 (0.0) 0 (0.0)	11 (25.0) 22 (50.0)	3 (6.8) 5 (11.4)	1 (2.3) 2 (4.5)	0.975	9 (20.5) 16 (36.4)	6 (13.6) 13 (39.5)	0.759
Lymphocyte infiltration Yes No	3 (42.9) 4 (57.1)	0 (0.0) 0 (0.0)	2 (28.6) 4 (57.1)	1 (14.3) 0 (0.0)	0 (0.0) 0 (0.0)	0.212	2 (28.6) 3(42.9)	1 (14.3) 1 (14.3)	0.809
Distant metastasis Yes No	5 (22.7) 17 (77.3)	0 (0.0) 0 (0.0)	3 (13.6) 9 (40.9)	1 (4.5) 8 (36.4)	1 (4.5) 0 (0.0)	0.127	4 (18.2) 11 (50.0)	1 (4.5) 6 (27.3)	0.519
Tumor recurrence Yes No	14 (36.8) 24 (63.15)	0 (0.0) 0 (0.0)	11 (28.9) 16 (42.1)	3 (7.9) 7 (18.4)	0 (0.0) 1 (2.6)	0.619	7 (18.4) 16 (42.1)	7 (18.4) 8 (21.1)	0.311

* H-score*:histological score

Values in bold are statistically significant

*TNM stage and histological grade are defined only in squamous cell carcinoma (SCC) type.

### Survival outcomes in patients with melanoma and non-melanoma skin cancers (NMSC)

Information on survival outcomes of melanoma skin cancer tissues and non-melanoma patients is demonstrated in Table 4 in detail. As listed in the table, out of 33 melanoma patients, follow-up data was lost for five patients, and the remaining patients were followed for a mean duration of 39 months (min = 5, max = 138). In this interval, 24 (72.7%) patients experienced recurrence, and 19 (57.6%) patients died due to melanoma. In NMSC patients, follow-up data were available for 38 patients being followed for a mean duration of 41.26 months (min = 7, max = 80). Fourteen (19.2%) patients experienced tumor recurrence, and two (2.7%) patients died due to cancer-related complications.

**Table 4:** The main characteristics of patients enrolled for survival analysis in melanoma skin cancer tissues and non-melanoma skin cancer

Features	Melanoma skin cancer tissues N (%)	Non-melanoma skin cancer tissues N (%)
Number of patients (N)	28	38
Range of follow-up duration for DSS or PFS (months)	(5-138), (5-116)	(7–80), (1–80)
Mean duration of follow-up time for DSS or PFS (months) (SD)	39 (35.4), 22.1 (22.5)	41.26 (26.3), 33.68 (24.2)
Median duration of follow-up time for DSS or PFS (months) (Q1, Q3)	22 (16-52.7), 12.5 (12–24)	31 (19–67), 24 (18.7–61.7)
Cancer-related death (N %)	19 (57.6)	2 (2.7)
Distant metastasis during follow-up (N %)	22 (66.7)	5 (6.8)
Tumor recurrence during follow-up (N %)	24 (72.7)	14 (19.2)
Patients without distant metastasis and tumor recurrence (N %)	3 (9.1)	23 (31.5)

### Prognostic value of Talin-1 expression in melanoma and non-melanoma skin cancers (NMSC) patients

**Fig. 4:**
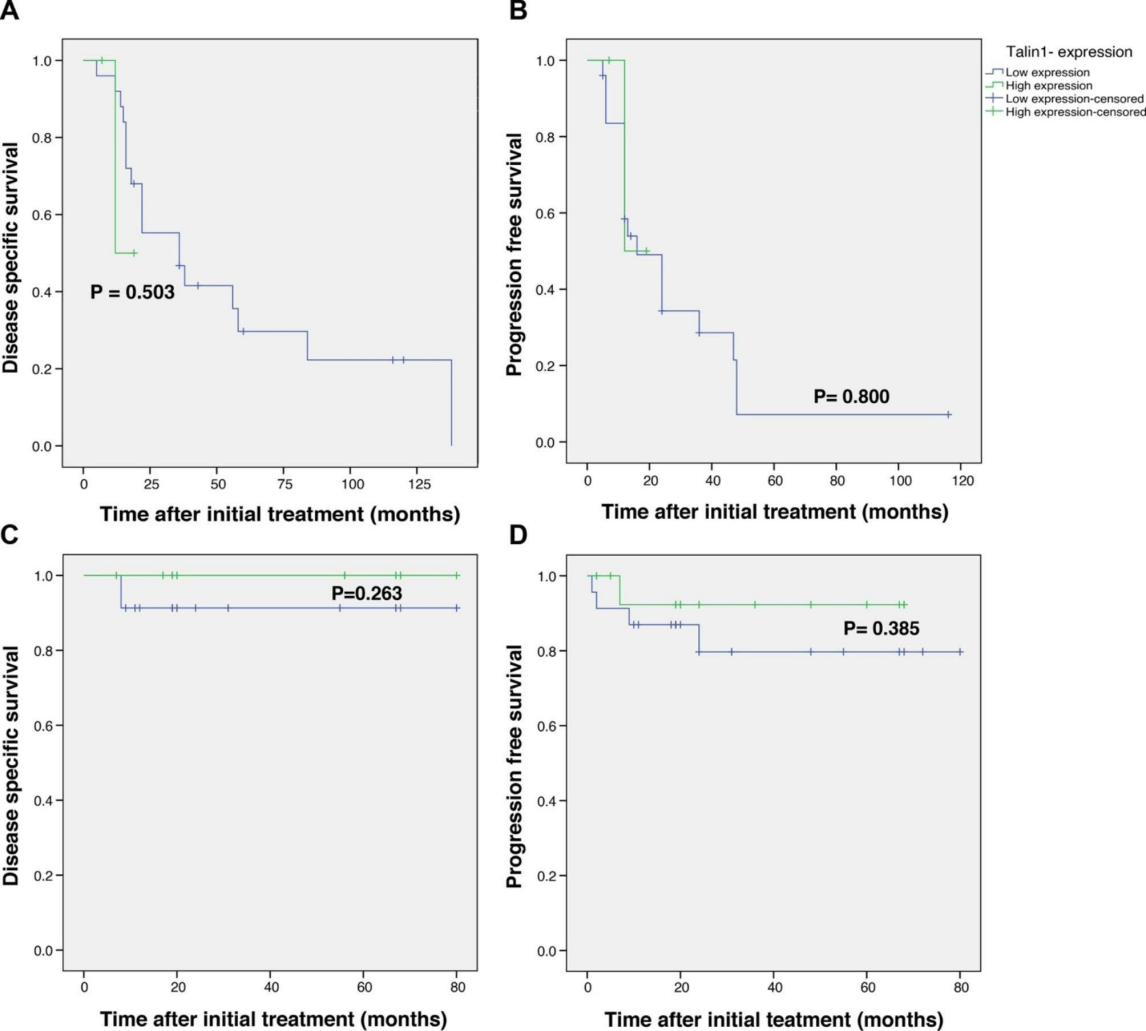
**Kaplan-Meier survival curves for disease-specific survival (DSS) and progression-free survival (PFS) based on Talin-1 cytoplasmic protein expression in skin cancer tissue samples.** The Kaplan-Meier survival curves showed no significant differences between DSS (A) or PFS (B) of melanoma skin cancer patients with high or low expression of Talin-1. Likewise, no significant differences were seen between DSS (C) or PFS (D) of non melanoma skin cancer (NMSC) patients with high or low expression of Talin-1. However, shorter DSS durations were seen in melanoma patients with higher levels of Talin-1

We used Kaplan–Meier survival analysis to compare DSS or PFS based on Talin-1 expression (H-score) in melanoma and NMSC patients. Our findings showed that no significant associations between DSS or PFS and the patients with high and low expression of the Talin-1 protein in melanoma (Log-rank test: DSS P = 0.503, PFS P = 0.800) and NMSC cases (Log-rank test: DSS P = 0.263, PFS P = 0.385). (Fig. 4).

Utilizing univariate and multivariate analyses, we assessed the clinical significance of various parameters that might influence DSS and PFS in these patients. The results indicated that the listed clinicopathologic variables are not significant factors affecting the DSS and PFS of melanoma and NMSC patients.

## Discussion

Considering the significant burden of skin cancers on public health, identification and characterization of molecular and cellular processes involved in oncogenesis and tumor progression are vital to uncovering novel prognostic markers and therapeutic targets [8, 9, 52]. In this regard, this study was conducted to evaluate the potential of Talin-1 protein as a biomarker of skin cancer. In in-silico analysis, using the bioinformatics approach, we explored the omics data and identified significantly dysregulated gene expression of Talin-1 in skin cancers. Additionally, in-silico data indicated increased expression of Talin-1 in metastatic tissues in mRNA level. Furthermore, experimental expression of Talin-1 protein through IHC method indicated a significant difference in cytoplasmic expression of this protein in melanoma and NMSC tumor cells compared to normal skin tissue. The expression of Talin-1 protein in melanoma invasion has not been investigated prior to our study, although many studies have reported the involvement of proteins associated with Talin-1 in melanoma progression [53].

Vinculin is one of the main proteins connecting Talin-1 to the actin cytoskeleton [54]. Vinculin stabilizes the actin-FA binding, and its underexpression promotes melanoma motility and metastasis, whereas its activation inhibits tumor growth and sensitizes the tumor to chemotherapy [55–59]. As one of the main molecules in Talin-1 dependent integrin signaling and FA assembly, FAK plays a substantial role in PI3K/AKT signaling pathway [54, 60, 61], which is an important oncogenic pathway and therapeutic target in melanoma and NMSCs[62, 63]. AKT mutations in melanoma cell lines were associated with reduced inhibition of FAK and increased brain metastasis [64]. Furthermore, phosphorylation and constitutive activation of FAK have been suggested as the mechanisms accountable for anchorage-independent phenotype resulting in melanoma metastasis [59, 65–67]. In order to gain stemness properties and invasive phenotype, malignant melanocytes and keratinocytes undergo epidermal-mesenchymal transition (EMT), resulting in loss of their E-cadherin adhesions and invasion [68–70]. As a critical signaling molecule of the cytoskeleton, Talin-1 regulates cadherin adhesions and may play a role in the EMT process of skin cancers [43, 71–73]. In our study, Talin-1 staining was nearly exclusive to the cytoplasm of the skin tumor cells, confirming the previous evidence regarding the expression and function of Talin-1 [40, 74]. Moreover, our results show that the staining was not limited to the epidermal-dermal junction unlike normal skin tissue [32]. These differences can be explained, in part, by considering the fact that disruption occurs by the tumor. Cancer-associated Talin-1 mutation and dysregulations induce metastasis by disrupting integrin activity, leading to the loss of cell adhesion and organization [75, 76].

The evaluation of the staining pattern in melanoma and NMSCs exhibited differential expression of Talin-1 protein with a range of intensities from weak to strong. Moreover, there was a statistically significant difference between the cytoplasmic expression of Talin-1 protein in melanoma and NMSCs tissues. These findings are in line with the fact that melanoma and NMSCs vary significantly in their oncogenesis and progression [77]. Therefore, there is an urgent need to investigate each type separately because each type of skin cancer could be related to different prognostic values and behaviors and effective to select the best therapeutic decisions.

In the current study, we observed a positive correlation between Talin-1 expression and LVI in melanoma skin cancer tissues. The LVI has been shown to be an independent prognostic factor increasing the risk of metastasis in melanoma [78]. Furthermore, higher expression levels of Talin-1 associated with an increase in the stage of melanoma, showing the probable potential of Talin-1 for risk assessment in melanoma patients. Our results highlighted upregulation of Talin-1 in melanoma progression and LVI. Previous evidence suggested that Talin-1 is a critical molecule in integrin activation, signaling pathway, and cell adhesion [29, 79–81]. Per our results, in the literature, the association of Talin-1 upregulation with invasive cancer phenotype and higher stages of gastric and prostate cancer as well as nasopharyngeal carcinoma and oral SCC has been reported [35, 40, 42, 82]. The mentioned evidence implies Talin-1 is a crucial player in the integrin activation process and may have a promotive effect on malignant melanocytes, which may be hijacked in invasive melanoma by upregulating its expression for tumor invasion and progression.

Compared to melanoma, NMSCs have a less aggressive nature and tend to be localized [8, 83]. Other than tumor grade in SCC specimens, we observed no significant correlations between Talin-1 expression and NMSC clinicopathological characteristics. Poor histological grading in cutaneous SCC is associated with tumor recurrence, metastasis, and invasive phenotype of the tumor [84, 85], and it was associated with Talin-1 upregulation in our study. Previously Lai et al. reported the association of Talin-1 upregulation with poorly differentiated oral SCC, however, the tumor microenvironment and pathogenesis vary significantly in oral and cutaneous SCC [86, 87]. This finding may suggest Talin-1 as a predictor of invasive SCC, although more studies are needed to conclude.

Previous investigations indicated dysregulation of Talin-1 is associated with patients’ survival outcomes in colorectal and prostate cancers and oral SCC and nasopharyngeal carcinoma [35, 38, 39, 88]. Our Kaplan-Meier curve results showed no significant association of Talin-1 expression with melanoma and NMSC patients’ survival. However, upregulation of Talin-1 was significantly associated with melanoma recurrence after tumor resection in our study. These results may be due to the small and lost-to-follow-up patient population.

More than half of the melanoma tumors harbor an activating mutation in BRAF, a serine/threonine kinase protein, which enhances tumor proliferation and invasion, hence targeted BRAF inhibitor therapies such as Vemurafenib have been developed with high efficacy [89]. On the other hand, Immune Checkpoint Inhibitor therapies such as Pembrolizumab and Nivolumab target the immune evasion mechanisms of the tumor [90–92]. Unfortunately, despite all the advancements in melanoma targeted therapy and immunotherapy, resistance to novel therapies remains a major clinical problem [93]. The molecular mechanisms of resistance to immune checkpoint inhibitors (ICIs) and BRAF inhibitors (BRAFi) are mostly unknown, but the role of cytoskeletal remodeling and myosin reactivation is well established [94, 95]. Many studies have reported the change in the cellular shape of the BRAFi resistant sublines of melanoma, making them more fibroblast and spindle-like [96–100]. Rho GTPase is the main molecule responsible for the contraction of the actin cytoskeleton, cellular shape, and resistance to BRAFi therapy [101]. Rho GTPase is activated by DLC-1, which inhibits the actin contraction and promotes the Talin-1 refolding [24, 102]. Furthermore, the Yes-associated protein (YAP) pathway has been shown to regulate actin remodeling in BRAFi resistant cell lines via accumulation of YAP in the nucleus [98]. Talin-1 unfolding leads to its binding to vinculin and translocation of YAP to nucleus and further adhesion growth [103]. Talin-1 can alter the myosin-driven machinery via DLC-1 binding [54]. The loss of DLC-1 protein function in melanoma tissue samples significantly promotes melanoma’s aggressiveness and deteriorates patients’ prognosis [54, 104]. The changes in the actomyosin skeleton render resistant melanoma cells highly dependent on cytoskeletal signaling pathways [95]. Furthermore, mutations in integrin signaling pathways improve melanoma patients’ outcomes after ICI therapy [105]. Considering the substantial role of Talin-1 in cytoskeletal and integrin signaling pathways, presuming a pivotal role for Talin-1 in response to ICI and BRAFi therapy is highly probable and warrants further investigations in the future.

As the evidence in other cancers implies, Talin-1 may have a presumptive effect on skin cancer patients’ survival outcomes, and more studies are needed to establish the prognostic value of Talin-1 in skin cancers. A limitation of our study was the small patient population, which may have restricted our observations. Therefore, a larger sample can lead to more generalizable results.

## Conclusion

In conclusion, our data mining analysis indicated upregulation of Talin-1 in skin cancer patients in mRNA level in comparison with normal skin tissues. Our finding from protein evaluation also demonstrated increased expression of Talin-1 protein in skin cancer tissues compared to normal tissues. Moreover, our results exhibited differential expression of Talin-1 protein between melanoma and NMSCs tissues with a statistically significant difference between the two groups, which may affect their prognosis and treatment options. Furthermore, overexpression of Talin-1 was associated with higher stages, local invasion, and recurrence of melanoma, emphasizing the role of cytoskeletal adhesion and signaling in melanoma progression and invasion. Our findings suggest Talin-1 may have a presumptive effect on prognosis. Therefore, further studies are needed on the function of Talin-1 as a potential biomarker in skin cancers as well as its prognostic value.

## Electronic supplementary material

Below is the link to the electronic supplementary material.

Supplementary Material 1

Supplementary Material 2

Supplementary Material 3

Supplementary Material 4

## Data Availability

The datasets used and/or analyzed during the current study are available from the corresponding author on request.

## List of abbreviations

NMSC: Non-Melanoma Skin Cancer
FFPE: Formalin-Fixed Parafin Embedded
TMA: Tissue MicroArray
IHC: ImmunoHistoChemistry
SCC: Squamous Cell Carcinoma
BCC: Basal Cell Carcinoma
LDH: Lactate Dehydrogenase
FAK: Focal Adhession Kinase
FA: Focal Adhession
DLC-1: Deleted in Liver Cancer-1
ECM: Extracellular Matrix
GEPIA: Gene Expression Profiling Interactive Analysis
GENT2: Gene Expression database of Normal and Tumor tissues 2
TCGA: The Cancer Genome Atlas
GTEx: Genotype-Tissue Expression
GDC: Genomic Data Commons
H&E: Hematoxylin and eosin
LVI: LymphoVascular Invasion
PNI: Perineural Invasion
DSS: Disease-Specific Survival
PFS: Progression-Free Survival
AJCC: American Joint Committee on Cancer
TBS: Tris-Buffered Saline
DAB: 3,3′-diaminobenzidine
H-Score: Histochemical Score
SD: Standard Deviation
EMT: Epidermal-Mesenchymal Transition
ICI: Immune Check-point Inhibitor
