# Supplementary material for: High expression of Talin-1 is associated with tumor progression and recurrence in melanoma skin cancer patients
Source: BMC Cancer. 2023 Apr 3;23:302. doi: 10.1186/s12885-023-10771-z (PMC10069040; doi:10.1186/s12885-023-10771-z)
Supplement: Supplementary file 1 — Supplementary Material 1 [file 12885_2023_10771_MOESM1_ESM.docx]

| Supplementary Table 1. Investigation of Talin-1 on the Gene Expression database of Normal and Tumor tissues 2 )GENT2( database for skin cancer. | | |
| --- | --- | --- |
| Microarray platforms | P-value | Log_2_FC |
| GPL570 platform (HG-U133_Plus_2) | < 0.001 | 0.864 |
| GPL96platform (HG-U133A)] | 0.299 | -0.188 |
