# Supplementary material for: High expression of Talin-1 is associated with tumor progression and recurrence in melanoma skin cancer patients
Source: BMC Cancer. 2023 Apr 3;23:302. doi: 10.1186/s12885-023-10771-z (PMC10069040; doi:10.1186/s12885-023-10771-z)
Supplement: Supplementary file 2 — Supplementary Material 2 [file 12885_2023_10771_MOESM2_ESM.docx]

| Supplementary Table 2. Patients and the clinicopathological characteristics of melanoma skin cancer tissues. | |
| --- | --- |
| Patients and tumor characteristics | Melanoma skin cancer tissues N (%) |
| Number of patients | 33 |
| Mean age, years (Range)  ≤ Median age  > Median age | 45 (16-74)  18 (54.5)  15 (45.5) |
| Gender  Male  Female | 18 (54.5)  15 (45.5) |
| TNM stage  I  II  III  IV | 3 (9.1)  3 (9.1)  2 (6.1)  25 (75.8) |
| Breslow thickness (Range)  < 1  1- 4  4 > | 3 (21.4)  5 (35.7)  6 (42.9) |
| Ulceration  Yes  No | 10 (38.4)  16 (61.5) |
| Lymphovascular invasion (LVI)  Yes  No | 10 (58.8)  7 (41.1) |
| Perineural invasion (PNI)  Yes  No | 4 (16.6)  20 (83.3) |
| Lymphocytic infiltration  Yes  No | 12 (75.0)  4 (25.0) |
| Distant metastasis  Yes  No | 25 (75.8)  8 (24.2) |
| Tumor recurrence  Yes  No | 24 (85.7)  4 (14.3) |
