# Supplementary material for: High expression of Talin-1 is associated with tumor progression and recurrence in melanoma skin cancer patients
Source: BMC Cancer. 2023 Apr 3;23:302. doi: 10.1186/s12885-023-10771-z (PMC10069040; doi:10.1186/s12885-023-10771-z)
Supplement: Supplementary file 3 — Supplementary Material 3 [file 12885_2023_10771_MOESM3_ESM.docx]

| Supplementary Table 3. Patients and the clinicopathological characteristics of non-melanoma skin cancer (NMSC) tissues. | |
| --- | --- |
| Patient and tumor characteristics | Non-melanoma skin cancer tissues N (%) |
| Number of patients | 73 |
| Mean age, years (Range)  ≤ Median age  > Median age | 45 (16-74)  38 (52.1)  35 (47.9) |
| Gender  Male  Female | 57 (78.1)  16 (21.9) |
| TNM stage*  I  II  III  IV | 17 (43.6)  15 (38.5)  2 (5.1)  5 (12.8) |
| Histological grade*  Well  Moderate  Poor | 21 (53.8)  13 (33.3)  5 (12.8) |
| Ulceration  Yes  No | 15 (34.1)  29 (65.9) |
| Lymphocyte infiltration  Yes  No | 3 (42.9)  4 (57.1) |
| Distant metastasis  Yes  No | 5 (12.8)  34 (87.1) |
| Tumor recurrence  Yes  No | 14 (36.8)  24 (63.1) |
| *TNM stage and histological grade are defined only in squamous cell carcinoma (SCC) type. | |
