# Supplementary material for: High expression of Talin-1 is associated with tumor progression and recurrence in melanoma skin cancer patients
Source: BMC Cancer. 2023 Apr 3;23:302. doi: 10.1186/s12885-023-10771-z (PMC10069040; doi:10.1186/s12885-023-10771-z)
Supplement: Supplementary file 4 — Supplementary Material 4 [file 12885_2023_10771_MOESM4_ESM.pdf]

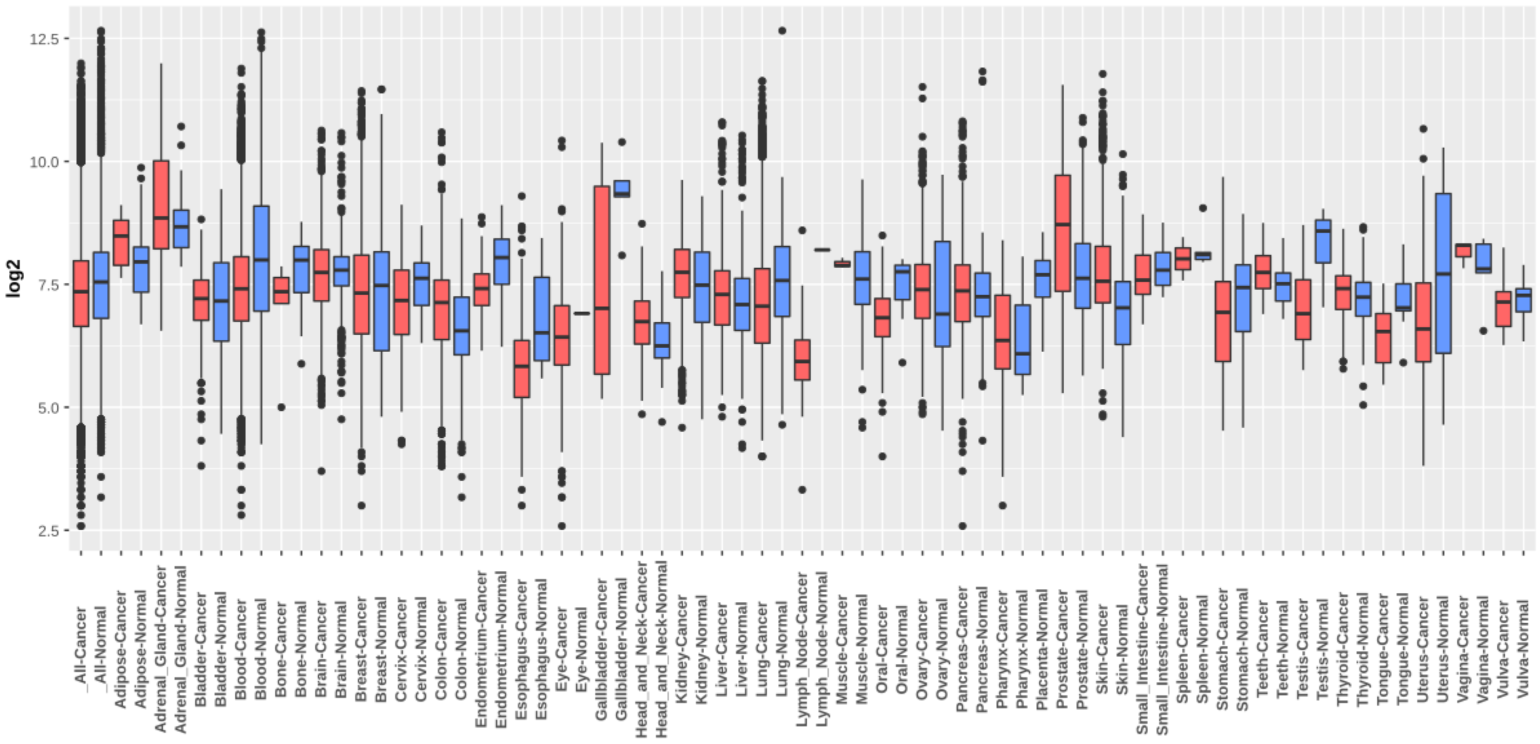

Supplementary Fig. 1. The mRNA levels of Talin-1 in skin cancers using Gene Expression database of Normal and Tumor tissues 2 (GENT2). Gene Expression Omnibus (GEO) mRNA expression level of Talin-1 was significantly higher in skin cancer (GPL570 platform) tissues compared to the normal tissues (|Log2FC| = 0.864 and P < 0.001).
